# Adipose Tissue-Derived Microvascular Fragments From Male and Female Fat Donors Exhibit a Comparable Vascularization Capacity

**DOI:** 10.3389/fbioe.2021.777687

**Published:** 2021-10-27

**Authors:** Thomas Später, Julia E. Marschall, Lea K. Brücker, Ruth M. Nickels, Wolfgang Metzger, Ann-Sophie Mai, Michael D. Menger, Matthias W. Laschke

**Affiliations:** ^1^ Institute for Clinical and Experimental Surgery, Saarland University, Homburg, Germany; ^2^ Department of Trauma, Hand and Reconstructive Surgery, Saarland University, Homburg, Germany

**Keywords:** tissue engineering, microvascular fragments, gender, vascularization, angiogenesis, dermal substitute, dorsal skinfold chamber

## Abstract

Adipose tissue-derived microvascular fragments (MVF) represent effective vascularization units for tissue engineering. Most experimental studies exclusively use epididymal fat tissue of male donor mice as a source for MVF isolation. However, in future clinical practice, MVF-based approaches may be applied in both male and female patients. Therefore, we herein compared the vascularization capacity of MVF isolated from the epididymal and peri-ovarian fat tissue of male and female donor mice. Freshly isolated MVF from male and female donors did not differ in their number, length distribution, viability and cellular composition. After their assembly into spheroids, they also exhibited a comparable *in vitro* sprouting activity. Moreover, they could be seeded onto collagen-glycosaminoglycan matrices, which were implanted into full-thickness skin defects within mouse dorsal skinfold chambers. Repetitive intravital fluorescence microscopy as well as histological and immunohistochemical analyses revealed a comparable vascularization and incorporation of implants seeded with MVF of male and female origin. Taken together, these findings demonstrate that the vascularization capacity of MVF is not gender-specific.

## Introduction

In tissue engineering, the function and survival of implanted tissue constructs crucially depends on their rapid and adequate vascularization, which ultimately ensures a sufficient oxygen and nutrient supply (Rouwkema and Khademhosseini, 2016). To achieve this, the seeding of implants with adipose tissue-derived microvascular fragments (MVF) has been suggested as a promising vascularization strategy over the last years (Laschke and Menger, 2015), which has been proven to be superior to single cell-based approaches (Später et al., 2018a).

MVF represent a randomized mixture of functional arteriolar, capillary and venular vessel segments, which can be easily isolated in large amounts from adipose tissue by means of mechanical dissection and enzymatic digestion (Frueh et al., 2017a; Später et al., 2017a; Frueh et al., 2017b). After their isolation, MVF exhibit an intact vessel morphology with a central lumen surrounded by endothelial cells and stabilizing pericytes (Laschke and Menger, 2015). Once seeded onto scaffolds and implanted into tissue defects, MVF rapidly interconnect with each other and the surrounding host vasculature. Thereby, their length of up to 150–200 μm allows them to bridge relatively wide distances (Später et al., 2017b). This, in turn, results in an early onset of blood perfusion in both peripheral and central areas of the implants (Shepherd et al., 2004).

Under experimental conditions, MVF are typically isolated from the epididymal fat pads of male donor mice or rats (Gruionu et al., 2010; Underwood et al., 2014; Frueh et al., 2017b; Später et al., 2020). However, in future clinical practice, MVF-based vascularization approaches may be applied in both male and female patients. In this context, it should be considered that ovarian hormones exert strong effects on the angiogenic activity and gene expression of endothelial cells (Kayisli et al., 2004; Yasuo and Kitaya, 2009) as well as on the mechanisms controlling blood flow and circulation (Sarrel, 1990). Accordingly, it is tempting to speculate that MVF isolated from adipose tissue of male and female donors may markedly differ in terms of their angiogenic potential and *in vivo* vascularization capacity.

To test this hypothesis, we isolated MVF from the epididymal and peri-ovarian fat pads of male and female donor mice and compared their number, length distribution, viability, cellular composition and sprouting activity. In addition, freshly isolated MVF of male and female origin were seeded onto collagen-glycosaminoglycan (CGAG) matrices, which were subsequently implanted into full-thickness skin defects within mouse dorsal skinfold chambers to analyze their vascularization and incorporation by means of intravital fluorescence microscopy, histology and immunohistochemistry throughout an observation period of 2 weeks.

## Materials and Methods

### Animals

Adipose tissue was isolated from male and female C57BL/6 wild-type mice as well as green fluorescent protein (GFP)^+^ mice (C57BL/6-Tg (CAG-EGFP)1Osb/J; The Jackson Laboratory, Bar Harbor, ME, United States) with a comparable age of 8.3 ± 0.7 months (male) and 8.9 ± 0.6 months (female). Moreover, their body weight was >30 g to guarantee large epididymal and peri-ovarian fat pads containing sufficient amounts of MVF for the seeding of matrices (Später et al., 2017a). Of interest, transgenic GFP^+^ mice allow the detection of all tissue except erythrocytes and hair due to a widespread GFP fluorescence (Okabe et al., 1997). Dorsal skinfold chambers were implanted in wild-type male and female C57BL/6 mice (Institute for Clinical and Experimental Surgery, Saarland University, Homburg, Germany) with an age of 4–6 months and a body weight of 24–28 g. The animals were housed under a 12 h day/night cycle and were fed ad libitum with water and standard pellet food (Altromin, Lage, Germany).

### Isolation of Microvascular Fragments

**FIGURE 1:**
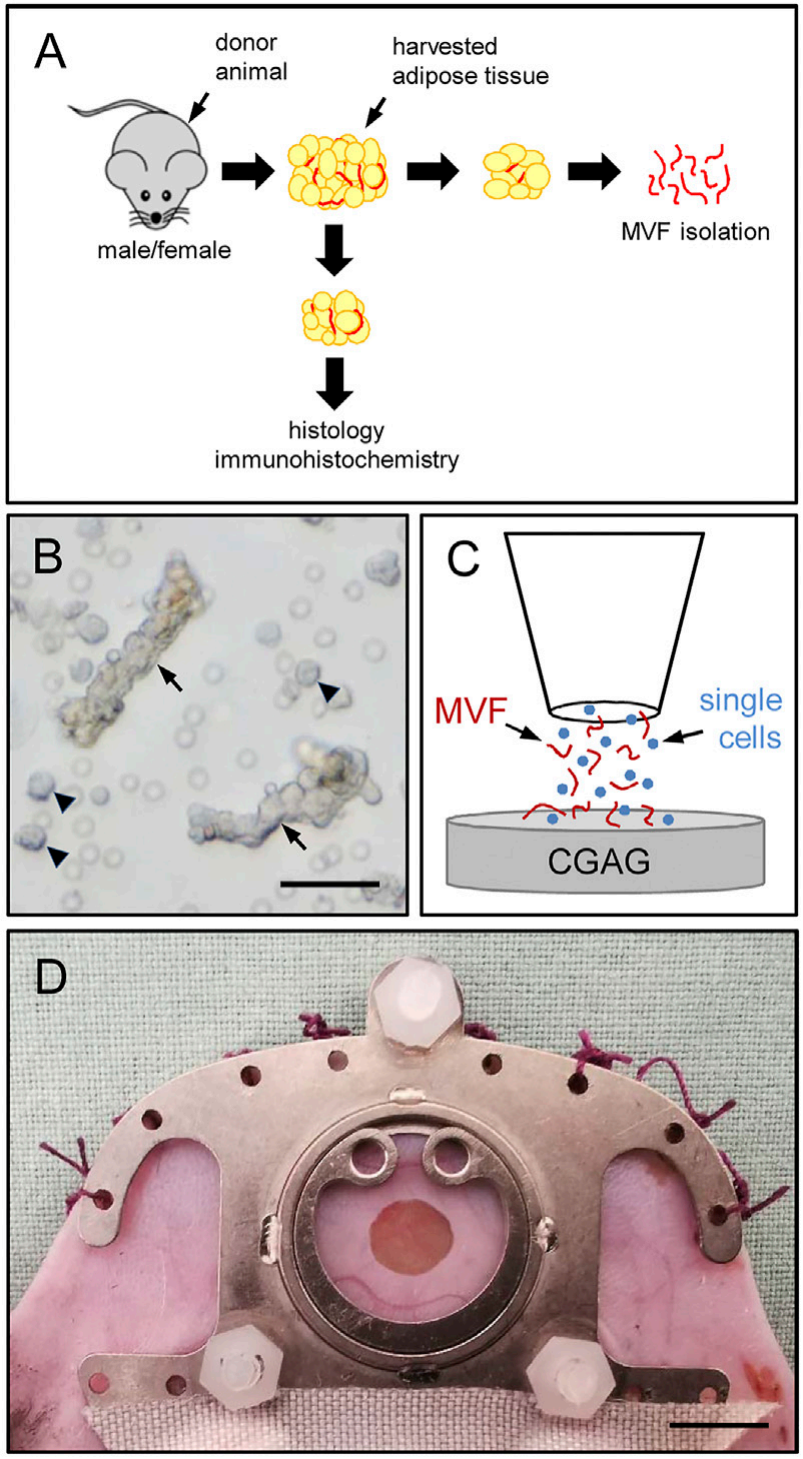
Experimental setting of the present study. **(A)** Epididymal and peri-ovarian fat pads were harvested from male and female donor mice. The adipose tissue was partly embedded for histological and immunohistochemical characterization or used for the isolation of MVF, which were used for *in vitro* and *in vivo* analyses. **(B)** Freshly isolated MVF (arrows) and surrounding single cells (arrowheads) from the epididymal fat pads of a male donor mouse. Scale bar: 50 µm. **(C)** Schematic illustration of the CGAG matrix seeding procedure. **(D)** Overview of an implanted dorsal skinfold chamber with observation window. Scale bar: 5 mm.

MVF were isolated from the epididymal and peri-ovarian fat pads of male and female donor mice as previously described (Frueh et al., 2017b) (Figure 1A). Briefly, the bilateral epididymal and peri-ovarian fat pads were transferred into 10% Dulbecco’s modified eagle medium (DMEM; 100 U/mL penicillin, 0.1 mg/mL streptomycin; Biochrom, Berlin, Germany) and washed thrice with phosphate-buffered saline (PBS). The isolated fat tissue was then mechanically minced and enzymatically digested with collagenase NB4G (0.5 U/mL; Serva Heidelberg, Germany) under slow stirring and humidified atmospheric conditions (37°C, 5% CO_2_) for 10 min. The digestion was neutralized with PBS supplemented with 20% fetal calf serum (FCS) and the cell-vessel suspension was subsequently incubated for 5 min at 37°C. After the fat supernatant was removed, the remaining suspension, which included both MVF and single cells, was filtered through a 300-µm mesh and the MVF (Figure 1B) were enriched to a pellet by a 5-min centrifugation at 120 *g*. The MVF pellet was either used for *in vitro* analyses or resuspended in 10 µL 0.9% NaCl for the seeding of CGAG matrices and subsequent *in vivo* analyses in the dorsal skinfold chamber model (Figures 1C,D). In addition, freshly isolated MVF were dispersed into single cells for flow cytometric measurements.

### Number, Length Distribution and Viability of Isolated Microvascular Fragments

At the end of each isolation process, both the number of individual MVF per mL adipose tissue and their length distribution were determined using a counting chamber and light microscopy. To further analyze the viability of freshly isolated MVF, they were transferred into 1 mL PBS containing 2 mg/mL Hoechst 33342 and 1 mg/mL propidium iodide (PI) (Sigma-Aldrich, Taufkirchen, Germany) for 15 min to assess the percentage of PI^+^ dead cells in relation to all counted cells by means of fluorescence microscopy.

### Generation of Microvascular Fragments Spheroids

Freshly isolated MVF were used to generate spheroids by means of the liquid overlay technique for the *in vitro* analysis of their sprouting activity. For this purpose, 20 wells of a 96-well plate were each coated with 40 µL 1% agarose (Sigma-Aldrich) and stored at room temperature for 30 min. Subsequently, ∼ 1,000 freshly isolated MVF suspended in 150 µL DMEM supplemented with 10% (v/v) FCS were transferred into each well with a 100 µL pipette and cultivated on the non-adherent agarose surface under humidified atmospheric conditions (37°C, 5% CO_2_) for 5 days. This resulted in the formation of stable MVF spheroids, which were then carefully removed from the wells and either processed for scanning electron microscopic analyses of their surface topography or embedded into collagen gel for sprouting assays.

### Scanning Electron Microscopy

The morphology of MVF spheroids was characterized by means of scanning electron microscopy. For this purpose, the spheroids were fixated using 2 vol% glutardialdehyde (Science Services GmbH, Munich, Germany) in 0.1 M sodiumcacodylate buffer at pH 7.4 (Carl Roth GmbH & Co. KG, Karlsruhe, Germany) for 10 min at room temperature under slight movement and were then stored at 4°C for at least 24 h. After washing with 0.1 M sodiumcacodylate buffer, the spheroids were dehydrated by incubation in an ascending ethanol series under movement (70 vol%, 80 vol%, 90 vol%, 96 vol% and 100 vol%). Finally, dehydration was completed by washing in a mixture (50:50) of 100 vol% ethanol and hexamethyldisilazane (Carl Roth GmbH Co. KG) followed by washing in pure hexamethyldisilazane. Subsequently, the spheroids were covered with hexamethyldisilazane, which was allowed to evaporate overnight. After the transfer of the spheroids to conductive carbon adhesive tabs (Plano GmbH, Wetzlar, Germany), they were sputtered to make them conductive as a prerequisite for the analysis. Sputtering was done 3 x for 60 s with gold (SCD 005, Balzers Union, Balzers, Liechtenstein) followed by an additional sputtering with carbon (SCD 030, Balzers Union). The spheroids were then analyzed in a FEI XL 30 ESEM FEG scanning electron microscope (FEI, Hillsboro, OR, United States) under high vacuum conditions at an acceleration voltage of 5 kV in secondary electrons mode.

### Sprouting Assay

To determine the angiogenic potential of MVF spheroids, their sprouting activity was analyzed by means of light microscopy. For this purpose, MVF from 5 independent isolations were used to generate a total of 100 spheroids per group, which were suspended in a collagen solution and transferred into pre-warmed 24-well plates. After 45 min, DMEM supplemented with 10% (v/v) FCS, 100 U/mL penicillin and 0.1 mg/mL streptomycin was added on top of the hardened collagen solution. After 1, 2, 3, 4 and 5 days, the total length of all sprouts (given in mm) per spheroid was measured.

### Flow Cytometry

For flow cytometric analyses, isolated MVF were further digested in Accutase^®^ (BioLegend, Fell, Germany) for 20 min into single cells. The single cells were then analyzed for the expression of the monoclonal rat anti-mouse endothelial cell marker CD31-phycoerythrin (PE) (BD Biosciences, Heidelberg, Germany), the perivascular cell marker mouse anti-α-smooth muscle actin (SMA) (Thermo Fisher Scientific Inc., Waltham, MA, United States) and the monoclonal stromal/stem cell surface markers rat anti-mouse CD117-fluorescein isothiocyanate (FITC) (BD Biosciences), mouse anti-rat/mouse CD90-FITC (BioLegend) and hamster-anti-mouse CD29-FITC (BioLegend). Isotype identical rat IgG-PE or rat IgG-FITC (BD Biosciences), mouse IgG-FITC (BD Biosciences) and hamster IgG-FITC (BioLegend) served as controls. Noteworthy, for the detection of intracellular α-SMA, the cells were first fixated and permeabilized in Cytofix/Cytoperm™ solution (BD Biosciences) for 20 min. Additionally, cells were analyzed for the expression of the purified polyclonal sheep anti-mouse/human adipocyte marker adipocyte-specific adhesion molecule (ASAM) (R and D Systems, Wiesbaden, Germany) followed by a secondary donkey anti-sheep IgG-Alexa488 antibody (Molecular Probes, Eugene, OR, United States). Flow cytometric analyses were performed by means of a FACScan (BD Biosciences) and data were assessed using the software package CellQuest Pro (BD Biosciences).

### Seeding of Collagen-Glycosaminoglycan Matrices

A 4-mm biopsy punch (kaiEurope GmbH, Solingen, Germany) was used to identically cut 12.6 mm^2^ CGAG samples out of a 1.3-mm thick Integra^®^ dermal regeneration template single layer without silicone sheet (Integra Life Sciences, Ratingen, Germany). These samples were then placed on a 500-µm cell strainer and 10 µL 0.9% NaCl containing ∼10,000 MVF were transferred onto them with a 20 µL pipette (Eppendorf, Wesseling-Berzdorf, Germany).

### Modified Dorsal Skinfold Chamber Model

For the *in vivo* analysis of MVF-seeded CGAG matrices, a modified dorsal skinfold chamber model was used according to Sorg et al. (2007; 2009). First, the mice were anesthetized by intraperitoneal injection of ketamine (75 mg/kg body weight; Ursotamin^®^; Serumwerke Bernburg, Bernburg, Germany) and xylazine (25 mg/kg body weight; Rompun^®^; Bayer, Leverkusen, Germany). Subsequently, two symmetrical titanium frames (Irola Industriekomponenten GmbH and Co. KG, Schonach, Germany) were fixed on the extended dorsal skinfold as previously described in detail (Laschke and Menger, 2016). After a 2-days recovery period, the mice were anesthetized again and a full-thickness skin defect (4 mm in diameter) was created within the observation window of the dorsal skinfold chamber by means of a dermal biopsy punch (kaiEurope GmbH). The defect was filled with a MVF-seeded CGAG matrix before the observation window of the chamber was sealed with a removable cover glass.

### Stereomicroscopy

To determine both epithelialization and implant-induced hemorrhage formation of MVF-seeded CGAG matrices by means of planimetry, the anesthetized animals were fixed on a Plexiglas stage and the dorsal skinfold chamber was positioned under a stereomicroscope (Leica M651, Wetzlar, Germany) on day 0 (day of implantation), 3, 6, 10 and 14. *Trans*-illumination was used to evaluate the extent of bleeding induced by the implants (given in % of implant surface) by means of a semiquantitative hemorrhagic score as follows: 1: No bleeding, 2: 1–25%, 3: 26–50%, 4: 51–75%, 5: 76–100%, 6: Bleeding exceeding implant surface. Furthermore, the chamber tissue was visualized in epi-illumination to identify epithelialized and non-epithelialized areas. The epithelialized area (given in %) was then calculated by the equation: (Total implant area—non-epithelialized implant area)/(total implant area) * 100. All microscopic images were recorded by a DVD system and analyzed by means of the computer-assisted off-line analysis system CapImage (Zeintl, Heidelberg, Germany).

### Intravital Fluorescence Microscopy

Following stereomicroscopy, 0.1 mL of the blood plasma marker 5% FITC-labeled dextran (150,000 Da; Sigma-Aldrich) was retrobulbarily injected into the venous plexus of the anesthetized animals for contrast enhancement. The observation window of the chamber was positioned under a Zeiss Axiotech microscope (Zeiss, Oberkochen, Germany) and the microscopic images were recorded by a charge-coupled device video camera (FK6990; Pieper, Schwerte, Germany) and a DVD system for off-line analyses by means of CapImage (Karschnia et al., 2018).

The vascularization of implanted CGAG matrices was assessed in 12 regions of interest (ROIs). ROIs exhibiting red blood cell (RBC)-perfused microvessels were defined and counted as perfused ROIs (in % of all ROIs) (Später et al., 2017a; Später et al., 2017b). Furthermore, the functional microvessel density (FMD) was determined as the total length of all RBC-perfused microvessels per ROI (given in cm/cm^2^). In addition, the diameter (d, given in µm) and centerline RBC velocity (v, given in µm/s) of 40 randomly selected microvessels were measured. Subsequently, these two parameters were used to calculate the wall shear rate (y, given in s^−1^) by means of the Newtonian definition y = 8 x v/d.

### Histology and Immunohistochemistry

Formalin-fixed tissue specimens were embedded in paraffin and cut into 3 µm-thick sections. Hematoxylin and eosin (HE) staining of individual sections was performed according to standard procedures. By using a BX60 microscope (Olympus, Hamburg, Germany) and the imaging software cellSens Dimension 1.11 (Olympus), the cross diameter of individual adipocytes (given in µm) and the density of adipocytes (given in mm^−2^) within epididymal and peri-ovarian fat pads were measured in 20 randomly selected ROIs. Moreover, the density of infiltrating cells (given in cells/mm^2^) was assessed in 12 randomly selected ROIs throughout each MVF-seeded CGAG matrix implanted in the dorsal skinfold chamber. Further sections were stained with Sirius red to quantify the collagen content within the implants as described previously in detail (Frueh et al., 2017a).

Additional sections were co-stained with a monoclonal rat anti-mouse antibody against CD31 (1:100; Dianova, Hamburg, Germany) and a polyclonal goat antibody against GFP (1:200; Rockland Immunochemicals, Limerick, PA, United States), while a goat anti-rat IgG Alexa555 antibody (Life Technologies, Ober-Olm, Germany) and a biotinylated donkey anti-goat antibody (1:30; Dianova) served as secondary antibodies. The biotinylated antibody was detected by streptavidin-Alexa488 (1:50; Life Technologies) and cell nuclei were stained with Hoechst 33342 (2 μg/mL; Sigma-Aldrich). This staining was used to analyze the density of CD31^+^ microvessels (given in mm^−2^) within freshly harvested epididymal and peri-ovarian fat pads as well as implanted MVF-seeded CGAG matrices and to assess the fraction of CD31^+^/GFP^+^ microvessels (given in %).

### Experimental Protocol

In a first set of experiments, epididymal and peri-ovarian fat pads were harvested from 10 male and 10 female C57BL/6 wild-type donor mice. The adipose tissue was partly embedded for histological and immunohistochemical characterization or used for the isolation of MVF and the assessment of their number per mL adipose tissue, length distribution, viability and *in vitro* sprouting activity.

In a second set of experiments, MVF were isolated from the epididymal and peri-ovarian fat pads of 8 male and 8 female GFP^+^ donor mice and seeded onto CGAG matrices (n = 8 per group). The seeded CGAG matrices were then implanted into full-thickness skin defects within dorsal skinfold chambers of 16 GFP^−^ wild-type C57BL/6 recipient mice (n = 8 per group). The vascularization, incorporation, bleeding and epithelialization of the implants were analyzed by means of repetitive stereomicroscopy and intravital fluorescence microscopy on day 0 (day of implantation), 3, 6, 10 and 14. Thereafter, the mice were sacrificed by means of cervical dislocation and the dorsal skinfold chamber preparations were processed for histological and immunohistochemical analyses.

### Statistical Analysis

After testing the data for normal distribution and equal variance, differences between the groups were analyzed by an unpaired Student´s *t*-test (SigmaPlot 11.0; Jandel Corporation, San Rafael, CA, United States). In case of non-parametric data, a Mann-Whitney rank sum test was used. All values are expressed as mean ± standard error of the mean (SEM). Statistical significance was accepted for a value of *p* < 0.05.

## Results

### Characterization of Epididymal and Peri-ovarian Adipose Tissue

**FIGURE 2:**
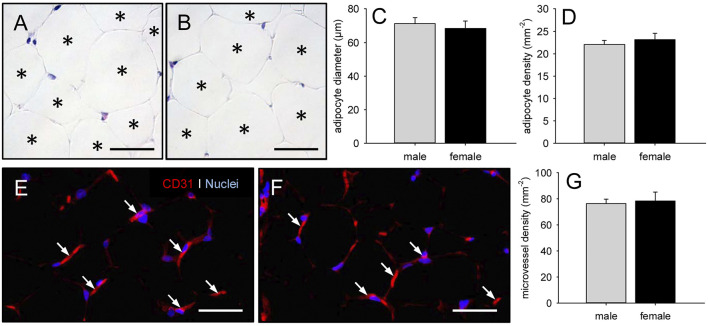
Characterization of epididymal and peri-ovarian adipose tissue. **(A, B)** HE-stained sections of freshly excised epididymal (A) and peri-ovarian (B) adipose tissue (asterisks = individual adipocytes). Scale bars: 50 µm. **(C, D)** Adipocyte diameter (C, given in µm) and adipocyte density (D, given in mm^−2^) within epididymal and peri-ovarian adipose tissue of male (gray bars, n = 3) and female (black bars, n = 3) C57BL/6 donor mice. Means ± SEM. **(E, F)** CD31^+^ microvessels (arrows) within epididymal (E) and peri-ovarian (F) adipose tissue. Cell nuclei were stained with Hoechst 33342. Scale bars: 40 µm. **(G)** Microvessel density (given in mm^−2^) within epididymal and peri-ovarian adipose tissue of male (gray bars, n = 3) and female (black bars, n = 3) C57BL/6 donor mice. Means ± SEM.

To assess the quality of the adipose tissue for MVF isolation, we first analyzed the epididymal and peri-ovarian fat pads of male and female C57BL/6 donor mice by means of histology and immunohistochemistry. This analysis showed no significant differences in terms of adipocyte diameter and density between the adipose tissue of male and female origin (Figures 2A–D). In addition, both fat tissue types exhibited a comparable microvessel density (Figures 2E–G).

### Characterization of Isolated Microvascular Fragments

**FIGURE 3:**
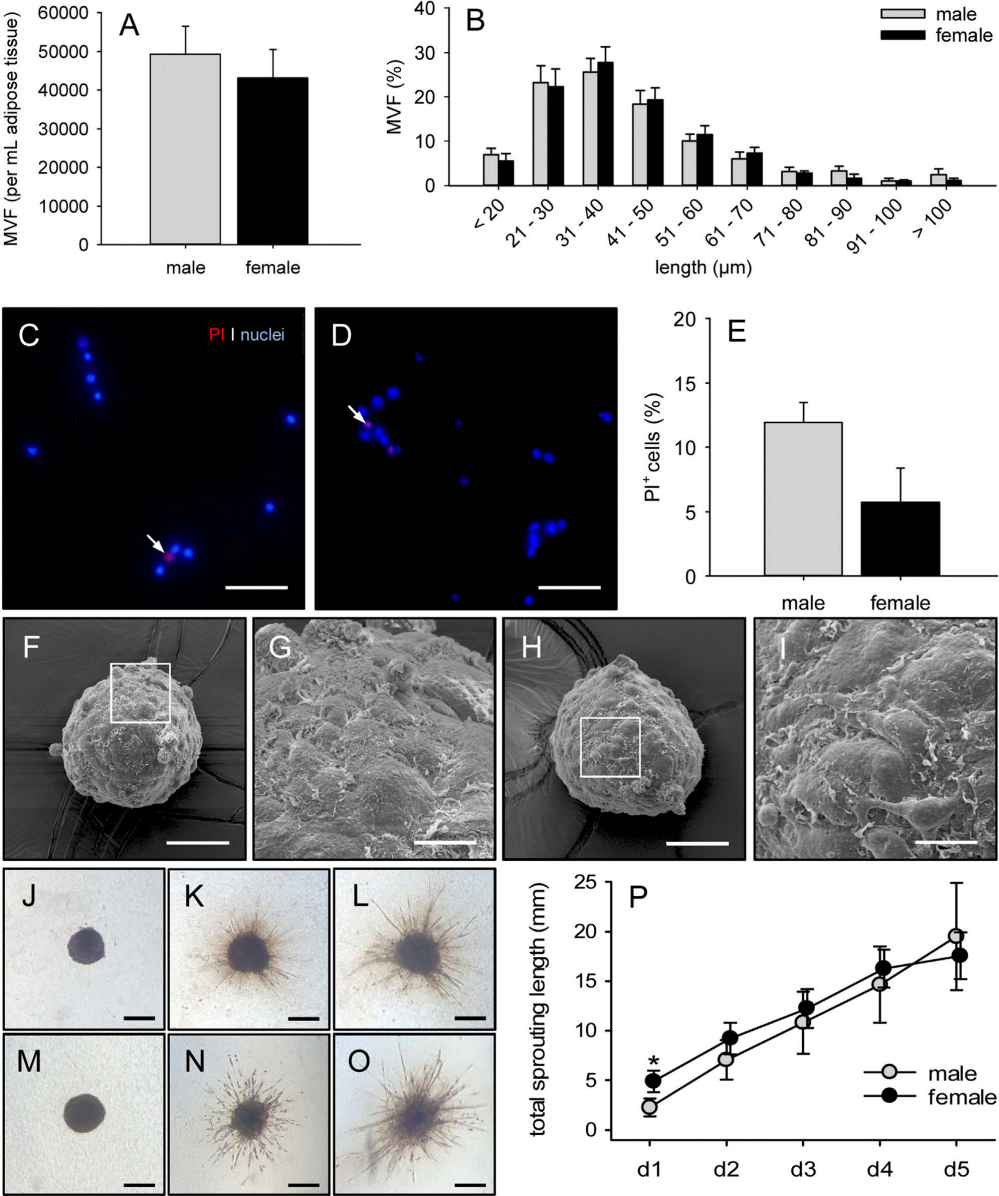
Characterization of isolated MVF. **(A, B)** Number (A, given as per mL adipose tissue) and length distribution (B, given in %) of MVF isolated from the epididymal and peri-ovarian fat pads of male (gray bars, n = 10) and female (black bars, n = 10) C57BL/6 donor mice. Means ± SEM. **(C, D)** Fluorescence microscopy of PI-stained MVF isolated from the epididymal and peri-ovarian fat pads of a male (C) and a female (D) C57BL/6 donor mouse for the assessment of viability (arrows = dead PI^+^ cells). Cell nuclei were stained with Hoechst 33,342. Scale bars: 60 μm. **(E)** PI^+^ cells (given in % of all counted cells) of MVF isolated from the epididymal and peri-ovarian fat pads of male (gray bar, n = 10) and female (black bar, n = 10) C57BL/6 donor mice. Means ± SEM. **(F-I)** Scanning electron microscopic images of spheroids generated from MVF of male (F, G) and female (H, I) origin. G and I = higher magnifications of white frames in F and H, respectively. Scale bars: F, H = 50 μm, G, I = 13 µm. **(J-O)** Light microscopy of angiogenic sprouting from spheroids generated from MVF of male (J–L) and female (M–O) origin on day 0 (J, M), 3 (K, N) and 5 (L, O) after embedding into collagen gel. Scale bars: 225 µm. **(P)** Total sprouting length (given in mm) of spheroids generated from MVF of male (gray circles, n = 5) and female (black circles, n = 5) origin. Means ± SEM. *p < 0.05 vs. male.

It was possible to isolate ∼40,000–50,000 MVF from both 1 mL epididymal and 1 mL peri-ovarian adipose tissue of C57BL/6 donor mice (Figure 3A). The length distribution of these MVF did not differ between the two groups (Figure 3B). Most of them exhibited a length of 21–50 µm (Figure 3B). Moreover, PI stainings revealed a comparably low fraction of dead cells within freshly isolated MVF of male and female origin (Figures 3C–E).

To analyze the angiogenic activity of MVF *in vitro*, we performed a spheroid sprouting assay. For this purpose, MVF spheroids were generated by means of the liquid overlay technique. As demonstrated by scanning electron microscopy, these spheroids were roundly shaped and exhibited an unregular, but comparable surface topography (Figures 3F–I). After their embedding into collagen gel, spheroids originating from male and female MVF started to form angiogenic sprouts, which progressively elongated throughout the observation period of 5 days (Figures 3J–P). Notably, the initial total sprouting length was significantly increased in the group of spheroids with MVF of female origin on day 1 when compared to spheroids with MVF of male origin (Figure 3P). However, during the following days, the total sprouting length was comparable in the two groups (Figure 3P).

Additional flow cytometric analyses showed a comparable cellular composition of MVF isolated from epididymal and peri-ovarian adipose tissue (Table 1). As expected, they contained a mixture of CD31^+^ endothelial cells, α-SMA^+^ perivascular cells, ASAM^+^ adipocytes as well as cells positive for the stromal/stem cell surface markers CD29, CD90 and CD117.

**TABLE 1:** Cellular expression (%) of CD31, α-SMA, ASAM, CD29, CD90 and CD117 in MVF isolated from the epididymal and peri-ovarian fat pads of male and female C57BL/6 donor mice (n = 5 per group), as assessed by flow cytometric analysis.

	CD31	α-SMA	ASAM	CD29	CD90	CD117
male	17.0 ± 1.4	10.1 ± 2.8	11.1 ± 1.7	52.0 ± 1.7	11.9 ± 1.8	8.4 ± 1.6
female	17 9 ± 4.2	8.2 ± 1.8	16.5 ± 4.6	54.2 ± 5.7	11.5 ± 3.8	10.1 ± 3.3

Mean ± SEM.

### *In vivo* Vascularization of Microvascular Fragments-Seeded Collagen-Glycosaminoglycan Matrices

**FIGURE 4:**
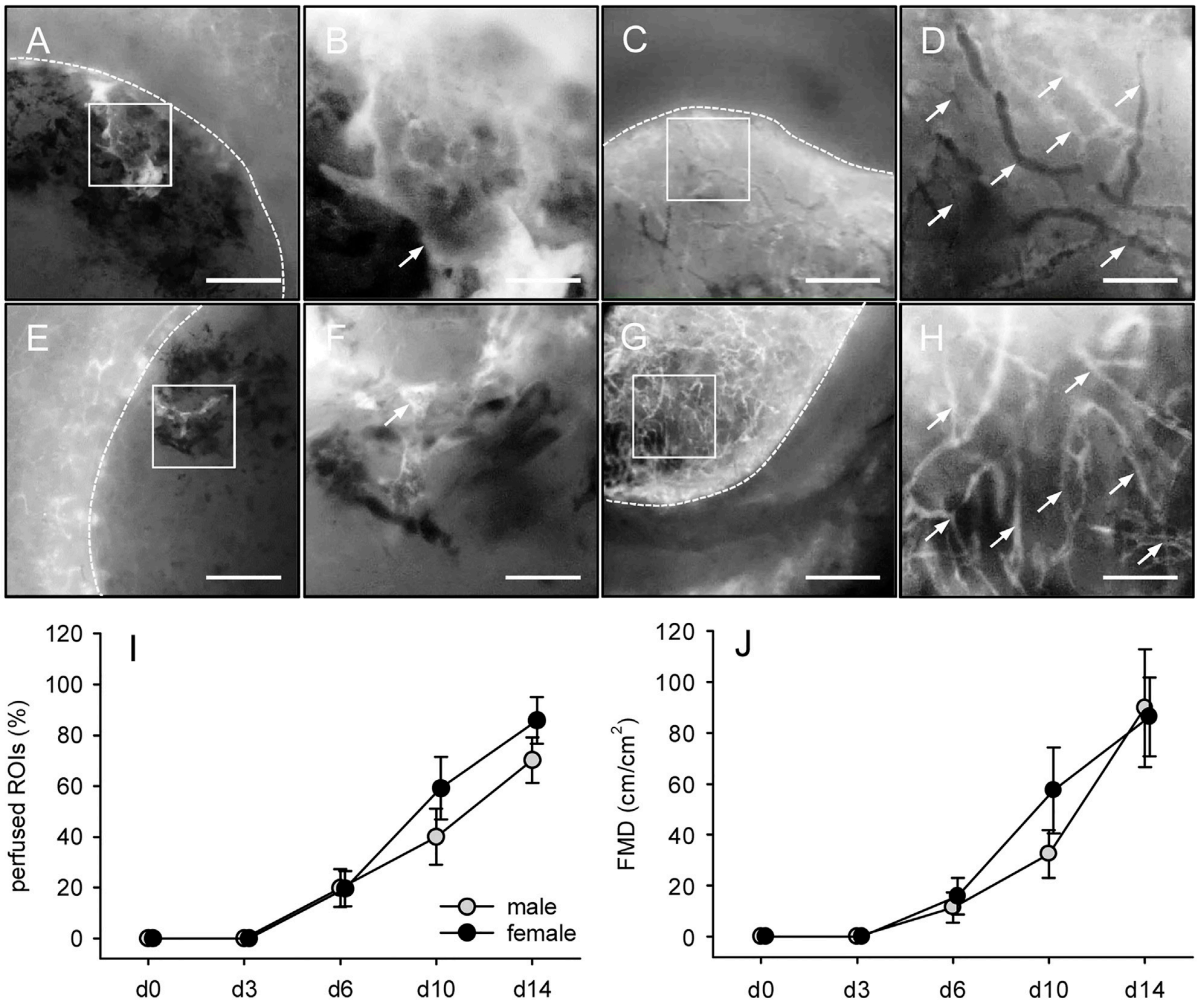
*In vivo* vascularization of MVF-seeded CGAG matrices. **(A-H)** Intravital fluorescence microscopy (blue light epi-illumination, 5% FITC-labeled dextran) of CGAG matrices (borders marked by broken lines) seeded with MVF of male (A–D) and female (E–H) origin on days 0 (A, B, E, F) and 14 (C, D, G, H) after implantation into full-thickness skin defects of male and female C57BL/6 recipient mice (arrows = blood-perfused microvessels). B, D, F and H = higher magnifications of white frames in A, C, E and G, respectively. Scale bars: A, C, E, G = 500 μm; B, D, F, H = 125 µm. **(I, J)** Perfused ROIs (I, given in %) and FMD (J, given in cm/cm^2^) of CGAG matrices seeded with MVF of male (gray circles, n = 8) and female (black circles, n = 8) origin on day 0, 3, 6, 10 and 14 after implantation into full-thickness skin defects of male and female C57BL/6 recipient mice, as assessed by intravital fluorescence microscopy and computer-assisted image analysis. Means ± SEM. *p < 0.05 vs. male.

To analyze the vascularization of MVF-seeded CGAG matrices *in vivo*, we used a modified dorsal skinfold chamber model. This model enabled the repetitive visualization and quantitative analysis of microvascular network formation within the implants by means of intravital fluorescence microscopy (Figures 4A–J). There were no marked differences in the *in vivo* vascularization capacity of MVF isolated from male and female fat samples. In both groups, first blood-perfused microvessels could be detected within the matrices on day 6 after implantation, indicating an early interconnection of individual MVF with the microvasculature of the surrounding host tissue (Figures 4A,B,E,F). Throughout the following time course, the seeded MVF reassembled into dense microvascular networks within the implants (Figures 4C,D,G–J). The maturation and remodeling of these networks was associated with decreasing diameters as well as increasing centerline RBC velocities and wall shear rates of individual blood vessels over time (Table 2).

**TABLE 2:** Diameter (µm), centerline RBC velocity (µm/s) and wall shear rate (s^−1^) of individual microvessels within CGAG matrices, which were seeded with MVF isolated from the epididymal and peri-ovarian fat pads of male and female GFP^+^ C57BL/6 donor mice (n = 8 per group), directly (d0) as well as 3, 6, 10 and 14 days after implantation, as assessed by intravital fluorescence microscopy and computer-assisted image analysis.

	0d	3d	6d	10d	14d
diameter (µm)					
male	—	—	24.9 ± 2.9	21.7 ± 2.0	15.7 ± 1.2
female	—	—	29.0 ± 1.5	25.8 ± 3.2	21.9 ± 2.5*
centerline RBC velocity (µm/s)					
male	—	—	194.2 ± 41.3	194.7 ± 34.4	205.9 ± 27.8
female	—	—	139.1 ± 34.2	238.2 ± 25.6	260.1 ± 30.2
wall shear rate (s^−1^)					
male	—	—	62.7 ± 17.8	83.0 ± 21.4	108.9 ± 19.0
female	—	—	38.8 ± 9.2	75.5 ± 5.5	101.6 ± 15.4

Mean ± SEM. *p < 0.05 vs. male.

### Implant-Induced Hemorrhage Formation

**FIGURE 5:**
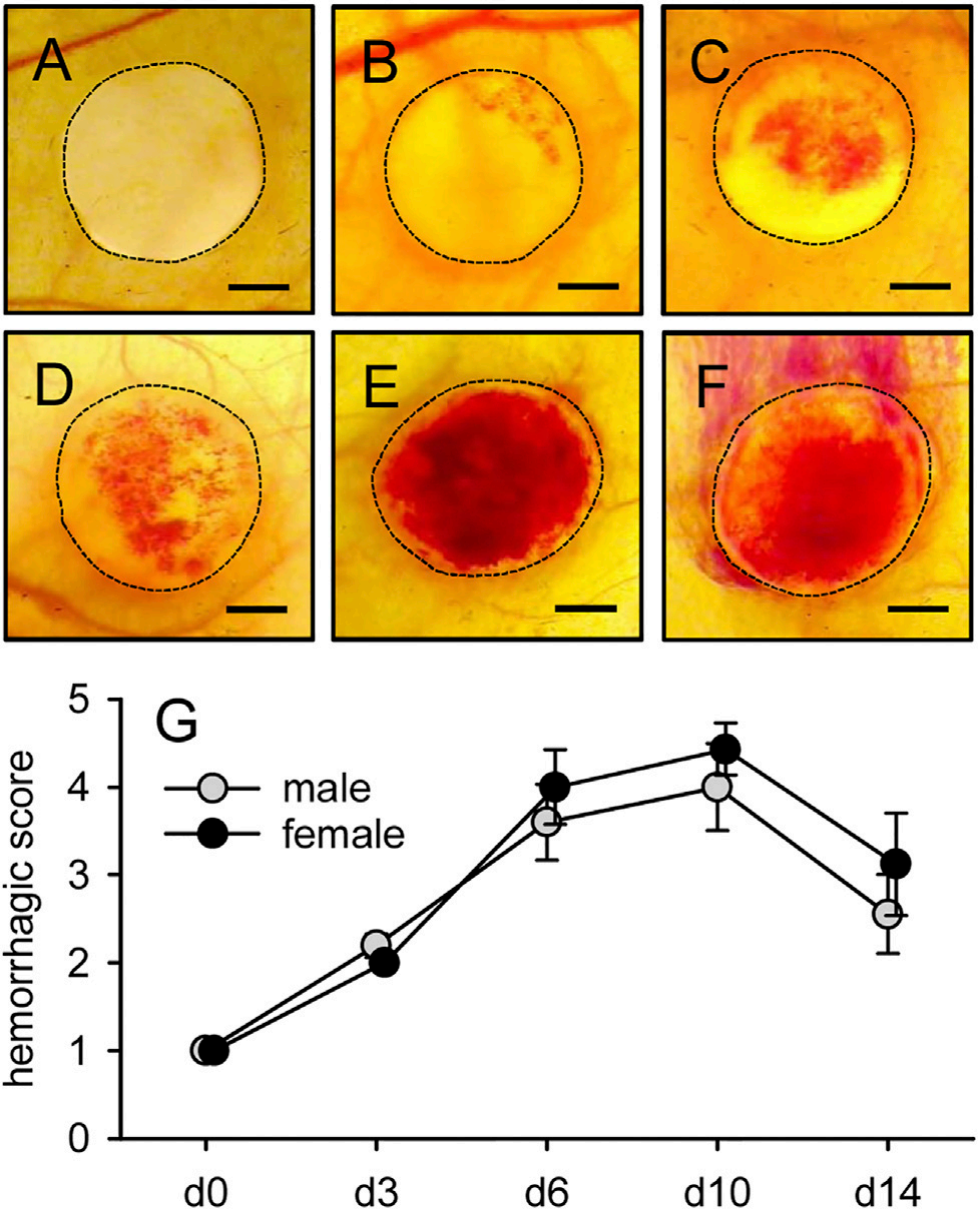
Implant-induced hemorrhage formation. **(A-F)** Stereomicroscopy in *trans*-illumination of implanted CGAG matrices seeded with MVF of male origin, displaying different manifestations of implant-induced hemorrhage formation according to a semiquantitative hemorrhagic score, i.e. 1: No bleeding (A), 2: 1–25% (B), 3: 26–50% (C), 4: 51–75% (D), 5: 76–100% (E), 6: Bleeding exceeding implant surface (F). Scale bars: 1.2 mm. **(G)** Hemorrhagic score of implanted CGAG matrices seeded with MVF of male (gray circles, n = 8) and female (black circles, n = 8) origin, as assessed by stereomicroscopy. Means ± SEM.

MVF-seeded CGAG matrices were additionally analyzed by means of *trans*-illumination stereomicroscopy to analyze implant-induced hemorrhage formation by means of a semiquantitative hemorrhagic score (Figures 5A–G). In line with the other *in vitro* and *in vivo* results, this score did not differ between matrices seeded with MVF of male and female origin. In both groups, the score progressively increased until day 10, reflecting hemorrhage formation during the early phase of implant vascularization, in which large parts of the newly developing networks within the implants were still incomplete and unorganized (Figures 4I, 5G). In contrast, the score decreased again towards the end of the 14-days observation period (Figure 5G).

### Incorporation and Vascularization of Microvascular Fragments-Seeded Collagen-Glycosaminoglycan Matrices

**FIGURE 6:**
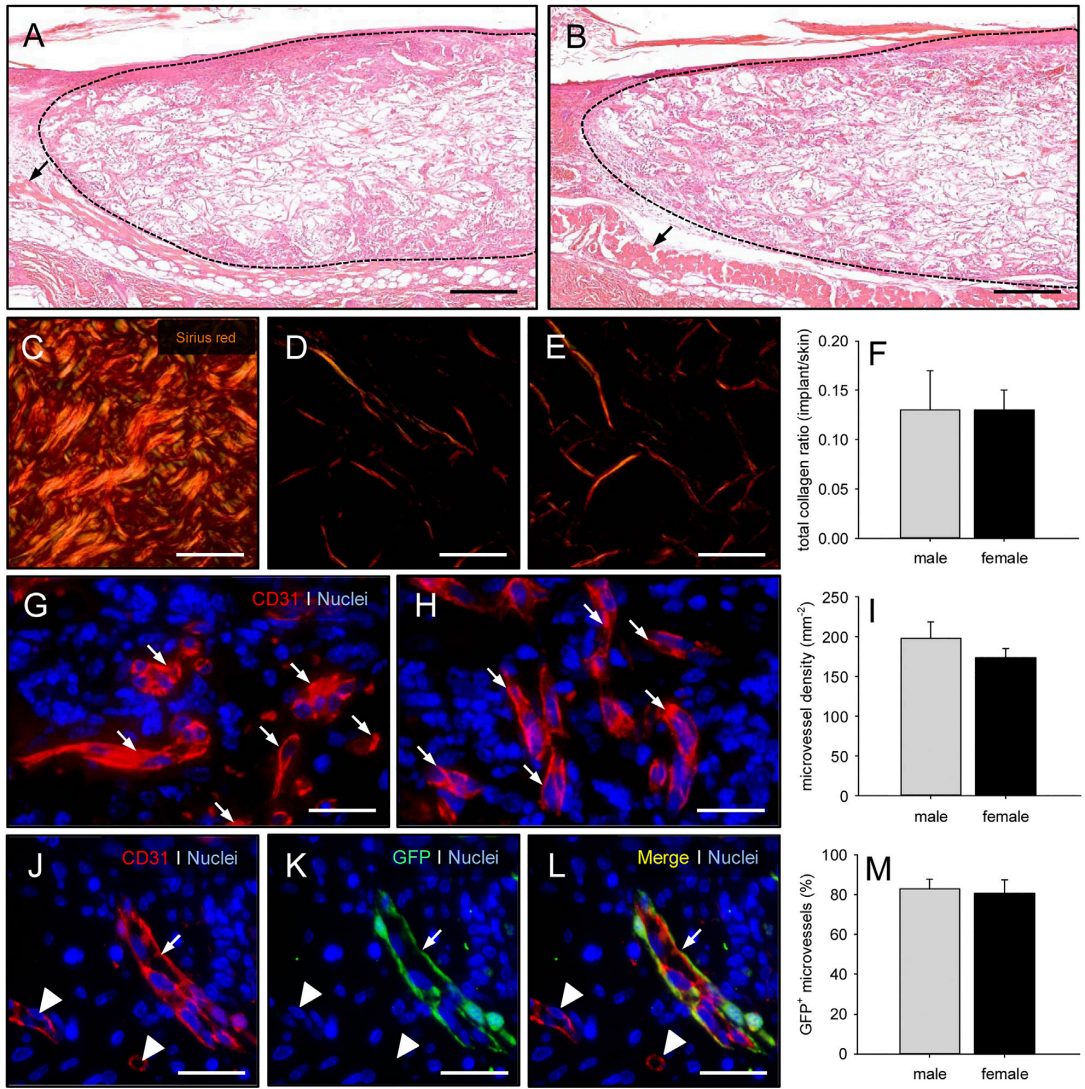
Incorporation and vascularization of MVF-seeded CGAG matrices. **(A, B)** HE-stained sections of CGAG matrices seeded with MVF of male (A) and female (B) origin 14 days after implantation into full-thickness skin defects of male and female C57BL/6 recipient mice (broken line = implant border; arrows = panniculus carnosus muscle). Scale bars: 230 µm. **(C-E)** Sirius red-stained sections of normal skin (C) as well as CGAG matrices seeded with MVF of male (D) and female (E) origin. Scale bars: 50 µm. **(F)** Total collagen ratio (implant/skin) of CGAG matrices seeded with MVF of male (gray bar, n = 8) and female (black bar, n = 8) origin. Means ± SEM. **(G, H)** Immunohistochemical detection of CD31 ^+^ microvessels (arrows) within CGAG matrices seeded with MVF of male (G) and female (H) origin. Scale bars: 45 µm. **(I)** Microvessel density (given in mm^−2^) of CGAG matrices seeded with MVF of male (gray bar, n = 8) and female (black bar, n = 8) origin. Means ± SEM. **(J-L)** Immunohistochemical detection of CD31^+^/GFP^+^ microvessels (arrows) within a CGAG matrix seeded with MVF of male origin (arrowheads = CD31^+^/GFP^−^ microvessels). Scale bars: 45 µm. **(M)** CD31^+^/GFP^+^ microvessels (given in %) within CGAG matrices seeded with MVF of male (gray bar, n = 8) and female (black bar, n = 8) origin. Means ± SEM.

At the end of the *in vivo* experiments, the implanted MVF-seeded CGAG matrices were additionally analyzed by means of histology and immunohistochemistry (Figures 6A–M). HE-stained sections revealed the infiltration of a dense granulation tissue into the implants of both groups. In fact, CGAG matrices seeded with MVF of male and female origin finally contained 4,666 ± 448 and 4,533 ± 577 cells/mm^2^, respectively. Further analyses of Sirius red-stained sections showed a comparable amount of mature type I collagen fibers within the implants (Figures 6C–F). Taken together, these findings indicate that the CGAG matrices were well incorporated into the full-thickness skin defects, independently of whether they were seeded with MVF from male or female donor mice.

In line with our *in vivo* results, additional immunohistochemical detection of the endothelial cell marker CD31^+^ demonstrated a comparable microvessel density in the matrices of both groups on day 14 after implantation (Figures 6G–I). CD31/GFP co-stainings further showed that approximately 80% of the detected microvessels were GFP^+^, indicating their origin from the seeded MVF of male or female origin (Figures 6J–M).

### Epithelialization of Microvascular Fragments-Seeded Collagen-Glycosaminoglycan Matrices

**FIGURE 7:**
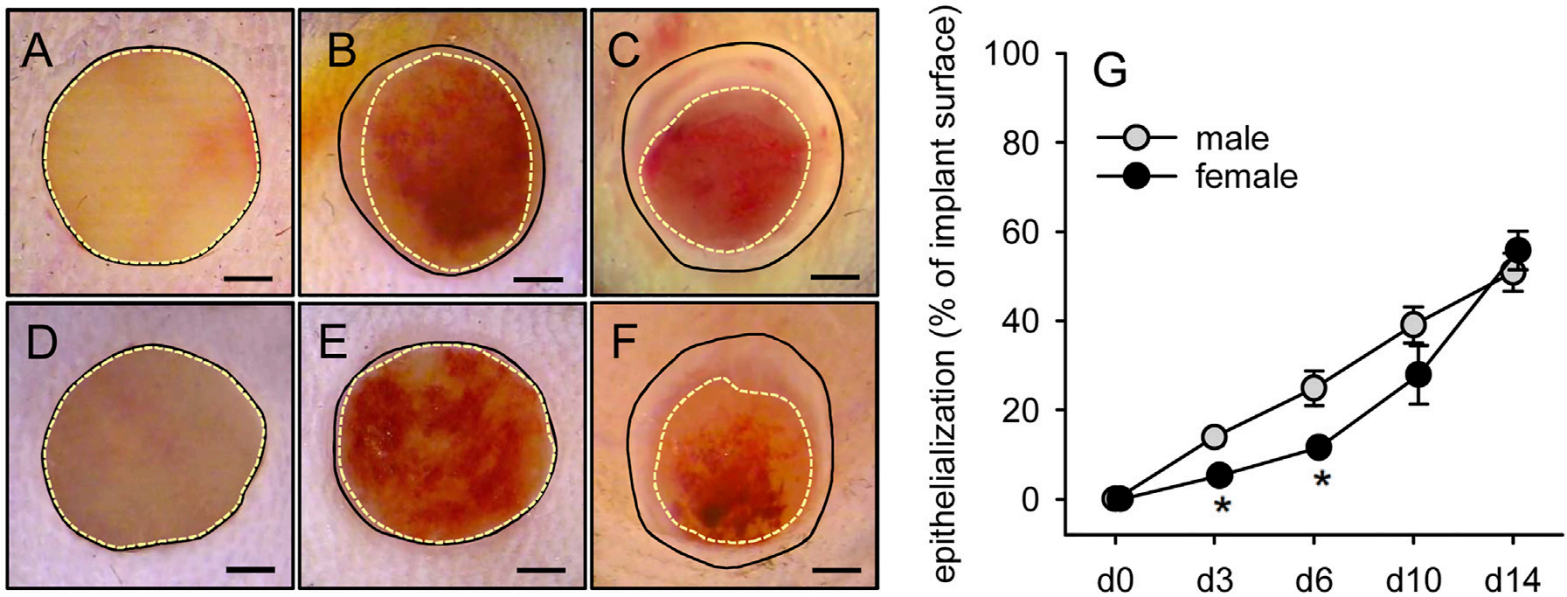
Epithelialization of MVF-seeded CGAG matrices. **(A-F)** Stereomicroscopy in epi-illumination of implanted CGAG matrices seeded with MVF of male (A–C) and female (D–F) origin on day 0 (A, D), 6 (B, E) and 14 (C, F) after implantation into full-thickness skin defects of male and female C57BL/6 recipient mice (closed lines = matrix borders, broken lines = non-epithelialized matrix areas). Scale bars: 750 µm. **(G)** Epithelialization (given in % of total implant surface) of CGAG matrices seeded with MVF of male (gray circles, n = 8) and female (black circles, n = 8) origin on day 0, 3, 6, 10 and 14 after implantation into full-thickness skin defects of male and female C57BL/6 recipient mice, as assessed by planimetric analysis of stereomicroscopic images. Means ± SEM. *p < 0.05 vs. male.

Finally, repetitive epi-illumination stereomicroscopy served to analyze the epithelialization of implanted MVF-seeded CGAG matrices over time (Figures 7A–F). Of interest, CGAG matrices seeded with MVF from female donors exhibited a significantly reduced epithelialization on days 3 and 6 after implantation when compared to implants seeded with MVF from male donors (Figure 7G). However, throughout the following time course of the experiment, the implants’ epithelialization did not differ anymore.

## Discussion

MVF exhibit a high angiogenic activity after their isolation from adipose tissue (Frueh et al., 2017a; Frueh et al., 2017b; Laschke et al., 2021). Accordingly, these fully functional vessel segments are commonly used in angiogenesis research to focus on basic mechanisms of microvascular network formation and the interaction of newly developing microvessels with the extracellular matrix in controlled three-dimensional environments (Kirkpatrick et al., 2007; Edgar et al., 2014; Utzinger et al., 2015). Moreover, MVF have been suggested as vascularization units for various applications in tissue engineering and regenerative medicine, such as the treatment of skin, muscle and bone defects (Pilia et al., 2014; Frueh et al., 2018; Orth et al., 2018). Until today, nearly all experimental studies exclusively used the epididymal adipose tissue of male mice and rats as the fat source for MVF isolation. This is due to the fact that male mice are known to accumulate more visceral fat tissue than female animals (Li et al., 2020). In addition, the epididymal fat pads are clearly defined and easy to harvest. However, under clinical conditions, both male and female patients should equally benefit from MVF-based vascularization approaches. In line with this view, we herein could demonstrate that MVF isolated from male and female adipose tissue exhibit a comparable vascularization capacity.

Since the yield and quality of isolated MVF may be markedly determined by the used fat source, we first characterized the epididymal and peri-ovarian adipose tissue of male and female C57BL/6 mice, which served as fat donors in the present study. It has previously been reported that the visceral adipose tissue of female mice contains higher levels of pro-angiogenic growth factors and develops a higher vascularity in response to high-fat diet (Rudnicki et al., 2018). In contrast, our results demonstrate that under physiological conditions both epididymal and peri-ovarian fat depots exhibit a comparable density of adipocytes and CD31^+^ microvessels. Accordingly, we could also isolate an identical number of MVF with a comparable length distribution from both tissue types. In line with previous studies (Später et al., 2017a), most of these MVF had a length of 21–50 µm. However, we also detected individual MVF of much larger sizes, which may particularly contribute to the rapid vascularization of seeded implants due the vessel segments’ ability to bridge large distances. In addition, we found that MVF of both male and female origin did not only contain CD31^+^ endothelial cells and α-SMA^+^ perivascular cells, but also many cells expressing the stromal/stem cell surface markers CD29, CD90 and CD117, which exhibit a high regenerative and angiogenic potential (Li et al., 2003; Li et al., 2005; Oishi and Ito-Dufros, 2006).

Recently, it has been shown that MVF can also be incorporated into spheroids without losing their native structure and cellular composition (Nalbach et al., 2021; Strobel et al., 2021). Moreover, spheroid sprouting assays are well-accepted *in vitro* tools for the analysis of angiogenesis within a physiological three-dimensional environment (Laschke and Menger, 2017; Zahra et al., 2019). Taking this into account, we herein generated MVF spheroids to analyze their angiogenic activity. For this purpose, we used the liquid overlay technique, which has previously been proven to be suitable for forming spheroids of standardized size (Metzger et al., 2011). By means of this approach, it was possible to generate stable MVF spheroids of both male and female origin within 5 days without any differences in the production process. These spheroids exhibited a comparable morphology with an unregular surface topography, which most probably reflects the heterogenous cellular composition of MVF. After their embedding into collagen gels, the initial total sprouting length was higher in the group of spheroids with MVF of female origin when compared to spheroids with MVF of male origin. This observation is in line with the fact that endothelial cells of female origin exhibit a higher proliferative and migratory activity (Boscaro et al., 2020). However, throughout the following days, the total sprouting length did not differ anymore between the two groups. Hence, potential gender-specific differences in endothelial cell activity and function did not markedly affect angiogenic sprout formation in our experimental setting.

In an additional set of *in vivo* experiments, we seeded freshly isolated MVF onto CGAG matrices, which are commonly used in clinical practice as dermal substitutes for the initial coverage of full-thickness skin defects (Chang et al., 2019). Moreover, we have already used these matrices in previous preclinical studies to assess the vascularization capacity of MVF under highly standardized conditions (Frueh et al., 2017a; Später et al., 2017a; Später et al., 2018c). For this purpose, we herein implanted MVF-seeded CGAG matrices into full-thickness skin defects within modified dorsal skinfold chambers of male and female recipient mice. Repetitive intravital fluorescence microscopic analyses revealed a comparable vascularization of implants seeded with MVF of male and female origin over an observation period of 14 days. The vascularization process did not only involve the reassembly of individual MVF into new microvascular networks but also their rapid interconnection with the surrounding vessels of the host tissue. The latter mechanism, also termed inosculation (Cheng et al., 2011), results in the early onset of blood perfusion, while the newly forming microvascular networks have not been fully established yet. Thus, it is associated with the leakage of blood and the formation of hemorrhages within the implants. In both groups, these hemorrhages were most pronounced between days 6 and 10 after matrix implantation. Thereafter, they receded, which can be explained by a progressive stabilization and organization of the microvascular networks as well as the simultaneous resorption of hemorrhages over time. Accordingly, we also detected a decline in diameters and an increase in centerline RBC velocities and wall shear rates of individual microvessels towards the end of the *in vivo* experiments, which is a typical sign for blood vessel maturation (Laschke et al., 2006).

In previous studies, we could already demonstrate that the vascularization of implanted CGAG matrices positively correlates with the implants’ incorporation into the surrounding host tissue (Frueh et al., 2017a; Später et al., 2017a). Such an incorporation into full-thickness skin defects is typically associated with the development of vascularized granulation tissue and collagen fiber formation over time (Später et al., 2018b). In line with our finding that CGAG matrices seeded with MVF of both male and female origin exhibited a comparable microvessel density on day 14 after implantation, none of these processes markedly differed between the two groups. In contrast, our stereomicroscopic analysis of the implants revealed a significantly reduced epithelialization of CGAG matrices seeded with MVF of female origin during the initial days after implantation. This is a surprising observation considering the fact that Kopcewicz et al. (2020) did not detect a major impact of gender on the re-epithelialization of cutaneous wounds in C57BL/6 mice. However, these wounds were not covered with a dermal substitute. Hence, it may be speculated that the different experimental setting in our study may have provoked this gender-specific deviation in the early epithelialization process.

Taken together, we could demonstrate that MVF of male and female origin exhibit a comparable vascularization capacity. This finding indicates that male and female mice can be equally used as fat donors in experimental MVF studies without the risk of significant gender-based biases. In addition, male and female patients may equally benefit from future clinical applications using MVF as vascularization units.

## Data Availability

The original contributions presented in the study are included in the article/Supplementary Material, further inquiries can be directed to the corresponding author.
